# BCG-induced trained immunity: history, mechanisms and potential applications

**DOI:** 10.1186/s12967-023-03944-8

**Published:** 2023-02-10

**Authors:** Jingjing Chen, Li Gao, Xinya Wu, Yuxin Fan, Meixiao Liu, Li Peng, Jieqin Song, Bingxue Li, Aihua Liu, Fukai Bao

**Affiliations:** 1 The Institute for Tropical Medicine, Kunming Medical University, Kunming, 650500 Yunnan China; 2 Yunnan Health Cell Biotechnology Company, Kunming, 650041 Yunnan China; 3 Department of Biochemistry and Molecular Biology, Kunming Medical University, Kunming, 650500 Yunnan China; 4 Department of Microbiology and Immunology, Kunming Medical University, Kunming, 650500 Yunnan China

**Keywords:** BCG, Trained immunity, Epigenetic reprogramming, Metabolic reprogramming, Viral infection, Cancer

## Abstract

The Bacillus Calmette-Guérin (BCG) vaccine was discovered a century ago and has since been clinically applicable. BCG can not only be used for the prevention of tuberculosis, but also has a non-specific protective effect on the human body called trained immunity that is mediated by innate immune cells such as monocytes, macrophages, and natural killer cells. Mechanisms of trained immunity include epigenetic reprogramming, metabolic reprogramming, and long-term protection mediated by hematopoietic stem cells. Trained immunity has so far shown beneficial effects on cancer, viral-infections, autoimmune diseases, and a variety of other diseases, especially bladder cancer, respiratory viruses, and type 1 diabetes. The modulation of the immune response by BCG has led to the development of a variety of recombinant vaccines. Although the specific mechanism of BCG prevention on diseases has not been fully clarified, the potential role of BCG deserves further exploration, which is of great significance for prevention and treatment of diseases.

Bacillus Calmette-Guérin (BCG) vaccine is an attenuated strain of *Mycobacterium bovis* obtained by serial passage. *Mycobacterium bovis* was firstly isolated in 1908 by Albert Calmette and Camille Guéri from a glycerol bile potato medium at the Pasteur Institute in Lille [1]. From 1908 to 1921, they serially passaged the strain and obtained a low-virulence strain and finally found that the strain protects the body from attack by the virulent *Mycobacterium tuberculosis*, and named it as BCG [1]. Trained immunity is the long-term functional reprogramming of innate immune cells, which is evoked by exogenous or endogenous insults, and leads to an increased effector function upon secondary stimulation after returning to an inactive state [2]. Compared to classical immunological memory, trained immunity has a number of characteristics. First, cells (myeloid cells, natural killer cells) and germline encoded recognition and effector molecules (e.g., pattern recognition receptors, cytokines) that differ from classical immunological memory are involved. Second, the increased responsiveness to secondary stimuli during trained immunity is not specific for a particular pathogen. Finally, trained immunity relies on changes in the functional state of innate immune cells that persists for weeks to months, rather than years, after the elimination of the initial stimulus [3]. In this review, we summarized the history, mechanisms, and potential applications of BCG-induced trained immunity.

## History of BCG-induced trained immunity

**Fig. 1:**
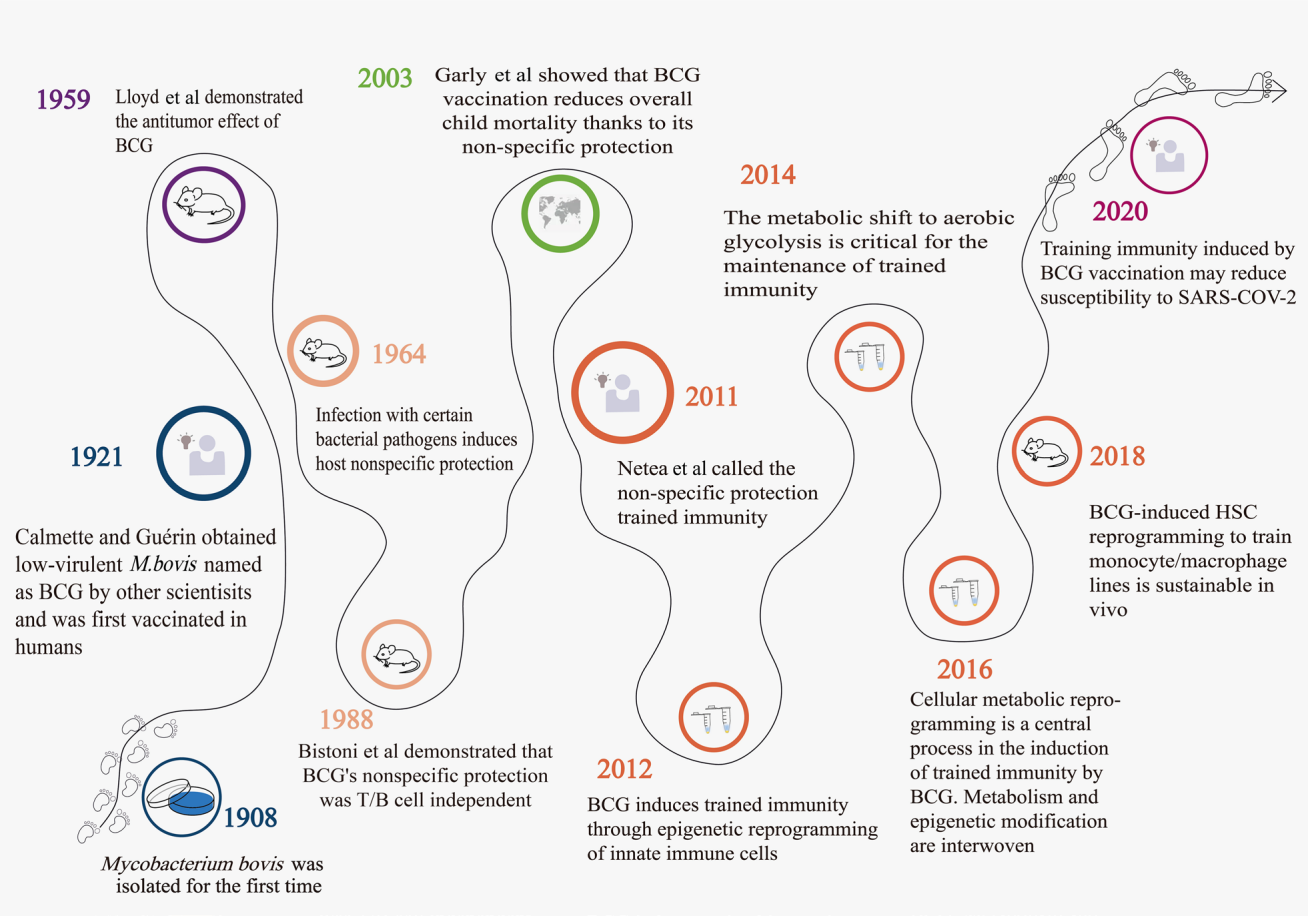
BCG and the history of trained immunity

BCG was first administered as a vaccine on 18 July 1921 at the Charité Hospital in Paris to an infant boy whose mother had died of tuberculosis [4]. Mass production of BCG began in 1924, and with the widespread vaccination of BCG through intradermal injection, its role was found to be more than just the prevention of tuberculosis. In 1928, Pearl found in an autopsy study that the incidence of cancer in tuberculosis patients was low [5], and subsequent epidemiological studies also showed that BCG could prevent child mortality independent of its effect on tuberculosis [6–8]. This sparked great interest and presented a new approach to studying the role of BCG in other diseases (Fig. 1).

The study by MacKaness GB et al. in 1964 showed that infection with certain bacterial pathogens would confer a high degree of resistance in the host against other unrelated pathogens, the host had nonspecific protection ("cross-protection"). It has been shown that BCG can induce host resistance to other infections [9, 10]. As further evidence for BCG induced non-specific protection, studies in the following years have also shown that BCG-inoculated mice can prevent infections such as *Plasmodium* [11], *Schistosoma manson* [12]. In 1988, Bistoni et al. elicited substantial protection against infection by *Candida albicans* vaccination in athymic mice, showing that cytotoxic T cells and B lymphocytes do not play a key role in the protection against *C. albicans* infections [13]. This protective effect, is independent of T/B cells, suggesting that BCG may exert a non-specific protective effect through a mechanism independent of adaptive immunity. In 2003, Garly et al. showed that BCG vaccination in West African children could reduce the morbidity caused by infections other than tuberculosis, thereby reducing the overall mortality, which profits from the non-specific protection of BCG vaccination [14]. There is a strong argument for BCG to induce non-specific protection against other infections, but the mechanism still requires further investigation.

Until 2011, studies had found that these nonspecific protective effects were mediated by innate immune cells such as monocytes (Mo), macrophages (Mφ), natural killer cells (NK), dendritic cells (DC), and neutrophils. Netea MG et al. showed that innate immunity conferred immune memory to innate host defenses. The feature is called “trained immunity” [3, 15]. A 2012 study, which combined in vivo and in vitro experiments, demonstrated that a NOD2-mediated epigenetic change at the level of histone methylation (H3K4me3) is the mechanism through which BCG enhances innate immune responses [16]. Saeed et al. in 2014 demonstrated the importance of epigenetic regulation in monocyte-to-macrophage differentiation and trained immune pathways [17]. At the same time, Cheng et al. proved that the shift of metabolic pathways to oxygen glycolysis is critical for the maintenance of trained immunity [18]. Regarding the relationship between epigenetics and metabolism-induced trained immunity, the study in 2016 showed that cellular metabolic reprogramming is a central process of BCG-induced trained immunity; metabolism and epigenetic modification are interwoven; and positive feedback loops may enhance trained immunophenotypes [19]. In view of the problem that mature innate immune cells (e.g., monocytes) have a short lifetime in circulation compared to the duration of trained immunity [20], a study in 2018 showed that the introduction of BCG into bone marrow (BM) alters the transcriptional patterns of hematopoietic stem cells (HSCs) and multifunctional progenitor cells (MPPs), and the monocytes/macrophages trained by BCG-induced HSC reprogramming are sustainable in vivo and provides better protection [21], which further extends the trained immune mechanism to the level of hematopoietic progenitors.

## Mechanisms of trained immunity

**Fig. 2:**
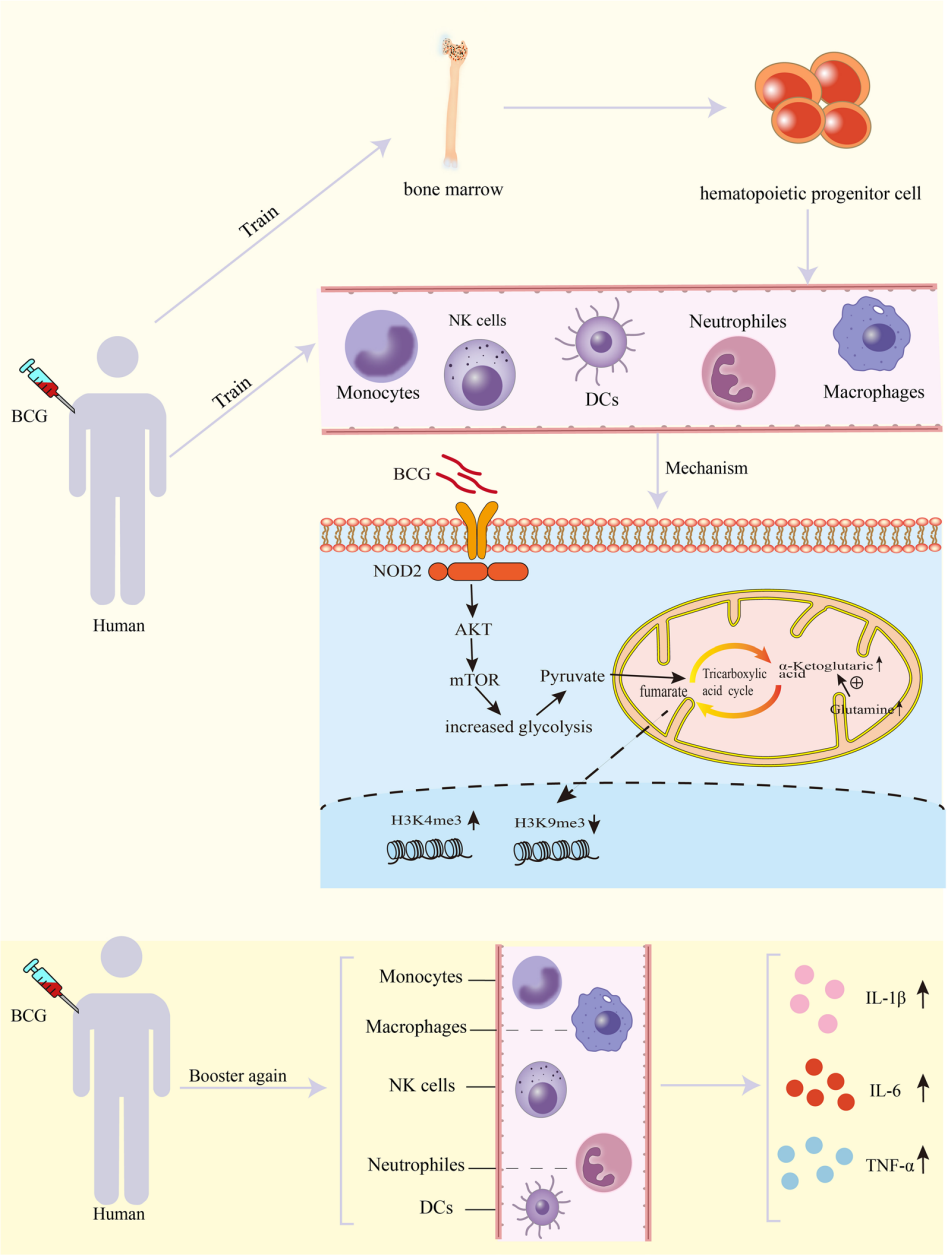
BCG induces trained immunity by binding to NOD2 receptors. The Akt/mTOR pathway is then activated for the metabolic switch to glycolysis. BCG induces epigenetic and metabolic reprogramming of innate immune cells to train innate immunity. Epigenetic reprogramming and metabolic reprogramming influence each other. Through the interaction with hematopoietic stem cells, it plays a long-term protective effect against infection. When the body was stimulated by BCG again, the cells after the training immunization produced more pro-inflammatory factors

Epigenetic reprograming is one of the molecular mechanisms that induces the development of trained immunity [22]. The different types of epigenetic modifications include DNA modifications, noncoding RNAs, histone modifications, and chromatin remodeling [23]. In addition to epigenetic reprogramming, different cellular metabolic pathways are also involved in the regulation and development of monocytes, macrophages, and NK cell-trained immunity. Epigenetic modification is a method of controlling gene expression that requires the coordination of cellular metabolism. Furthermore, epigenetic reprogramming is regulated by changes in immune cell metabolic flux. Trained immunity can play a long-term protective effect against infection, which is the result of interaction with hematopoietic stem cells [21] (Fig. 2).

### Epigenetic reprogramming

BCG induces trained immunity by binding to NOD2 receptors, and epigenetic recombination is the key to regulating gene expression to induce trained immunity [16]. Epigenetic mechanisms mainly include DNA methylation, post-translational modifications of histones, and noncoding RNA regulation. DNA methylation normally hinders transcription, whereas histone modifications can have more complex effects [24].

Epigenetic reprogramming of monocytes, characterized by deposition of chromatin marks and altered DNA methylation status, promotes the expression of pro-inflammatory genes, and metabolic reorganization underlies the long-term alterations in immune responses [19]. BCG vaccination induces histone modifications and epigenetic reprogramming in human monocytes at the promoter sites of genes encoding inflammatory cytokines such as TNF-α and IL-6 [16]. Trained monocytes and macrophages exhibited functional and epigenetic reprogramming, resulting in increased production of proinflammatory cytokines IL-6, IL-1, and TNF-α and chemokines, and enhanced phagocytosis and mortality [25]. At the same time, BCG also promotes NK cells to produce pro-inflammatory cytokines such as IL-1β and IL-6 [26]. Pro-inflammatory cytokines produced by cells, such as TNF-α, IL-1β and IL-6 coordinate local and systemic inflammatory responses. TNF-α and IL-1β sequentially activate the local endothelium, induce vasodilation, increase vascular permeability, and enable the recruitment of serum proteins and leukocytes to the site of infection. In addition, IL-1β, together with IL-6, activates hepatocytes to produce acute phase proteins. These proteins activate the complement and cause phagocytosis of pathogens by macrophages and neutrophils [27]. In addition, increased release of tumor necrosis TNF-α and IL-6 may prevent tuberculosis and viral infections [21, 28]. The reduction in viremia was highly correlated with the upregulation of IL-1β [28]. Enhanced neutrophil function persists for at least 3 months and is associated with genome-wide epigenetic modification of histone 3 trimethylation of lysine 4 (H3K4me3) [29]. Macrophages are trained to increase the expressions of various pattern recognition receptors (TLR4, CD206, and CD14); chemokine receptors (CCR2 and CXCR4); and costimulatory and/or signaling molecules (CD43, CD14, CD40) that correlated with chromatin remodeling marker H3K4me3 [30]. These receptors facilitate the stimulation of T cells, angiogenesis, and wound healing. As major players in the innate immune-triggered inflammatory response, inflammatory cytokines and chemokines play a key role in host defense against microbial infection. Inflammatory cytokines and chemokines play a crucial role in host defense against microbial infection [27, 31].

In addition to innate immune cells in local tissues, trained immunity has also been reported to be induced in myeloid progenitors of the BM, resulting in monocytes with higher immune potential and longer duration [21]. A recent study further demonstrated that BCG vaccination induces trained immunity through transcriptomic, epigenomic, and functional reprogramming of hematopoietic stem cells, progenitors and monocytes [32]. BCG may alter the BM microenvironment, leading to the production of cytokines via pathogen-associated molecular patterns (PAMPs), which may indirectly affect the function of hematopoietic stem and progenitor cells [21]. BCG can train innate immune cells to produce more cytokines, of which IL-1β has a strong effect on myelopoiesis [33]. IL-1β may be an endogenous mediator which links peripheral activation of monocytes and macrophages by BCG to long-term functional reprogramming at the level of myeloid progenitors.

### Metabolic reprogramming

Glycolysis, oxidative phosphorylation, and glutamine catabolism pathways were up-regulated in peripheral blood mononuclear cells after BCG inoculation [19]. Increased glycolytic metabolism in monocytes leads to a shift in the cellular metabolic program from oxidative phosphorylation to aerobic glycolysis (Warburg effect) [18]. Arts et al. [19] showed that BCG-induced trained immunity in monocytes was accompanied by a metabolic shift towards glycolysis through activation of the Akt/mTOR pathway. Metabolites not only provide substrates for biosynthesis but also regulate immune responses through signaling pathways and gene expression [34]. Most metabolites are important substrates and cofactors for epigenetic enzymatic activity.

Inhibited metabolism can reverse epigenetic changes in an in vitro trained immune model, resulting in reduced cytokine responses upon re-stimulation [35]. Healthy individuals taking metformin, an inhibitor of the mammalian target of rapamycin-dependent glycolysis, have reduced immune responses after training, through decreased histone H3 lysine 9 trimethyl marks (H3K9me3) [19].

## Potential applications

### Prevention and treatment of tuberculosis

The BCG has been used for prevention and treatment of tuberculosis for 100 years, but the effect of BCG varies from person to person. For children, BCG is mainly used to prevent disseminated tuberculosis [36]. The test conducted in Uganda from 1962 to 1970 showed that BCG had a major and sustained protective effect on the early childhood tuberculosis leprosy in this area [37]. In recent years, it has been found that after BCG is given to infant or school-age children after strict tuberculin testing, the lung tuberculosis can be prevented, and the protective effect of meningeal tuberculosis and millet tuberculosis seems to be greater than the protection of lung tuberculosis [38]. Therefore, early vaccination with BCG is beneficial to reducing the incidence of these diseases.

### Cancers therapy

BCG can also play a role against cancers. In 1959, Old LJ. et al. proved that the BCG had anti-tumor effect [39], and slowed the progress of the tumor by inducing immune stimulation effects [40]. In 1976, Morales A. et al. firstly used BCG to treat superficial bladder cancer [41]. Until now, BCG therapy is still the standard therapy for non-muscle invasive bladder cancer [42]. Although the study found that BCG treatment on the bladder cancer caused the poor sleep quality of patients with non-muscle invasive bladder cancer, effectiveness of BCG on bladder cancer is positive [43]. It is found that trained immunity level and antitumor effect may be improved by modifying BCG to express a higher level of key PAMP molecules [44]. This further proves the efficacy of BCG in the treatment of bladder cancer. In addition, BCG can also reduce the risk of melanoma [45], but also had used to the treatment of patients with melanoma in III stage [46]. The recent study showed that BCG can induce gastric cancer cell apoptosis and autophagy, activate lymphocytes, enhance anti-tumor activity of immune cells [47]. Moreover, the early BCG vaccination of children is associated with the risk of reducing lung cancer [48] and leukemia [49], and the earlier BCG vaccination is likely to reduce tumor mortality [50]. Another study showed that BCG may have an efficacy against kidney cancer and prostate cancer [51]. It can be seen that exploring the mechanism of the role of BCG is significant to the treatment of cancer.

### Antiviral effects

BCG can play an antiviral effect on a variety of viruses. For example, BCG-induced genome-wide epigenetic reprogramming in monocytes are proved to protect humans from experimental infections from yellow-fever attenuated vaccine strains, which may be critical for monocyte-produced IL-1β function to achieve a protective effect [28]. Since the body immunity of the elderly has different degrees of decline as the young, the elderly is easy to be infected by viruses, then the inoculation of BCG may be an effective prevention choice. The study has found that inoculating the elderly with BCG for 3 consecutive months, once a month, can significantly prevent their acute respiratory infections [52]. A mouse experimental study showed that BCG can prevent infections of various DNA and RNA viruses, including herpes and influenza viruses [53]. Moreover, in a randomized, placebo-controlled study, it is found that BCG vaccination before influenza vaccination can significantly improve the reaction amplitude and accelerated induction of the antibody against 2009 epidemic influenza A(H1N1), and the inoculation of BCG can also regulate the influence of the influenza vaccine on the production ability of cytokines [54]. The appearance of this phenomenon may be because BCG affects the humoral and cellular response to influenza vaccine. When influenza vaccine is administered, BCG in the body plays a certain strengthening role, which acts as an adjuvant and enhances the immune response of the body to influenza. Furthermore, BCG can also enhance the heterologous reactions against tetanus toxoid and poliovirus vaccines [55]. In a recent study, the recombinant BCG vaccine was constructed by inserting BZLF1 and LMP2 genes of EB viruses, rBCG vaccine expressing two genes exhibits an obvious effective immunosuppression for tumors of positive EB virus [56], which provides a good idea for continuing development of more effective antiviral vaccines.

For children’s common and flat warts, the local immunotherapy of BCG may be a new, effective, safe choice [57]. Nevertheless, different types of warts have different responses to BCG. A double-blind, randomized control study showed that intradermal injection of BCG is more effective in treating viral warts to a certain extent [58]. In addition, early BCG vaccination may be beneficial to relieve clinical syndrome and its long-term process of warts [59]. In summary, the time, the way and the dose of BCG vaccination have different effects on different diseases. Therefore, it is necessary to clarify the specific mechanism of BCG to prevent diseases.

In recent years, BCG has been found to have a huge potential against coronavirus Disease 2019 (COVID-19). Trained immunity induced by BCG vaccination during birth may have resistance to COVID-19 [60, 61]. First, when people are exposed to pathogens or pathogen components, they can strengthen BCG-induced trained immunity at birth, which is similar to re-vaccination, thus playing a role in resistance. Second, its resistance may be achieved through the inhibition of viral replication by BCG, resulting in a reduction in viral load, followed by a reduction in inflammation and symptoms. However, the protective effect of BCG was not found in another analysis [62]. In other words, although the people were inoculated with BCG vaccine, the analysis results may be different due to different characteristics of the population. Then BCG is insufficient to prevent COVID-19. Hence, the role of BCG on COVID-19 has no clear evidence, and further researches are needed to conduct.

### Treating autoimmune diseases

BCG may also have huge potential against autoimmune diseases. Studies have found that BCG inoculation is related to the reduction of hyperglycemia in patients with advanced type 1 diabetes [63]. The decrease of blood sugar is the joint action of the decrease in glucose production and the increase in glucose consumption. BCG vaccination can stabilize or even reduce the level of glycosylated hemoglobin, and induced TNF-α accelerate the death of islet self-reactive T-cells. In addition, the systematic transformation of glucose metabolism from oxidative phosphorylation to aerobic glycolysis showed a high utilization rate of glucose. Then the combined effect of the above mechanisms can achieve the goal of lowering blood sugar levels. The observations of autoimmune diabetic animal models are consistent with that since BCG has an immunomodulatory effect, perhaps it is connected with the decrease of positive autoantibodes of GAD65 and IA-2 in patients of South Indian diabetes [64]. In addition, in a mouse experiment, BCG infection can inhibit the development of experimental autoimmune encephalomyelitis [65]. BCG also can reduce the advancement of multiple sclerosis and retard the progress of brain lesions [66]. The above-mentioned studies have shown that BCG plays a major role in the treatment of autoimmune brain diseases. Interestingly, a cohort study showed that BCG did not affect the prevalence of asthma, eczema or pollen heat during children's period. However, neonatal vaccination BCG is associated with a low incidence of asthma [67]. This appears to be achieved by suppressing the immune response of helper Th2-type cells specific to asthma. At the same time, since BCG may change the microenvironment of the bone marrow, BCG may regulate the maturation of immune cells and the production of cytokines in the early stage or even in the whole process, thereby achieving long-term protective effects. A systematic review concluded that BCG vaccination might not represent an effective primary preventative strategy against the development of allergic sensitization and diseases [68]. Even so, there is still a study suggesting that BCG vaccination in the early days of life may prevent asthma by adjusting the immune maturation process [69]. Although there is no clear evidence for the effective effect of BCG on asthma, it is worth further study to clarify the value BCG on other autoimmune diseases.

### Other diseases and potential adverse effects

BCG may have certain effects on many other diseases. For instance, BCG's immunotherapy on bladder cancer is in connection with the significantly reduced risk of Alzheimer's disease and Parkinson's disease [70], and BCG reduces human malaria infection in a part of volunteers [71]. Surprisingly, there is insufficient evidence to prevent malaria by BCG vaccination [72, 73]. And BCG can promote the progression of chronic inflammatory diseases [74]. This shows trained immunity has beneficial effects on treating cancer, viruses, and autoimmune diseases, but trained immunity can also have harmful effects.

To sum up, although there is literature supporting the prote ctive effect of BCG induced trained immunity in some cases, the evidence in other infections and diseases is not always so strong.

## Conclusions

There is still much to learn about trained immunity. First, the mechanisms of trained immunity should be studied more deeply, including the molecular mechanisms and signaling pathways on the different cells involved, and further elucidation of metabolic reprogramming and epigenetic processes are needed. Second, to facilitate its application to more clinical diseases, it is necessary to clarify the effect of trained immunization on different diseases. Finally, the nonspecific protection of trained immunization can be used to develop a new generation of vaccines to realize the cross-protection potential of different vaccines.
